# Genetic variants of *TLR4*, including the novel variant, rs5030719, and related genes are associated with susceptibility to clinical malaria in African children

**DOI:** 10.1186/s12936-023-04549-8

**Published:** 2023-06-07

**Authors:** Amir Ariff, Yong Song, Ruth Aguilar, Augusto Nhabomba, Maria Nelia Manaca, Siew-Kim Khoo, Selma Wiertsema, Quique Bassat, Arnoldo Barbosa, Llorenç Quintó, Ingrid A. Laing, Caterina Guinovart, Pedro L. Alonso, Carlota Dobaño, Peter Le Souëf, Guicheng Zhang

**Affiliations:** 1 Centre for Genetic Origins of Health and Disease, The University of Western Australia and Curtin University, Perth, WA 6009 Australia; 2 School of Women’s and Children’s Health, University of New South Wales, Sydney, Australia; 3 School of Public Health, Curtin University, Perth, WA 6102 Australia; 4 ISGlobal, Hospital Clínic of Barcelona, Universitat de Barcelona, 08036 Barcelona, Spain; 5 Centro de Investigação em Saúde de Manhiça (CISM), 1929 Maputo, Mozambique; 6 ICREA, Pg. Lluís Companys 23, 08010 Barcelona, Spain; 7 Pediatrics Department, Hospital Sant Joan de Déu, Universitat de Barcelona, Esplugues, Barcelona, Spain; 8 Consorcio de Investigación Biomédica en Red de Epidemiología y Salud Pública (CIBERESP), Madrid, Spain; 9 Division of Cardiovascular and Respiratory Sciences, The University of Western Australia, Perth, WA 6009 Australia; 10 Telethon Kids Institute, The University of Western Australia, Perth, WA 6008 Australia; 11 School of Medicine, The University of Western Australia, Perth, WA 6008 Australia; 12 Curtin Health Innovation Research Institute, Curtin University, Perth, WA 6102 Australia

**Keywords:** Malaria, TLR4, Genetic polymorphisms, Haplotype trend regression, Africa, rs5030719

## Abstract

**Background:**

Malaria is a deadly disease caused by *Plasmodium* spp. Several blood phenotypes have been associated with malarial resistance, which suggests a genetic component to immune protection.

**Methods:**

One hundred and eighty-seven single nucleotide polymorphisms (SNPs) in 37 candidate genes were genotyped and investigated for associations with clinical malaria in a longitudinal cohort of 349 infants from Manhiça, Mozambique, in a randomized controlled clinical trial (RCT) (AgeMal, NCT00231452). Malaria candidate genes were selected according to involvement in known malarial haemoglobinopathies, immune, and pathogenesis pathways.

**Results:**

Statistically significant evidence was found for the association of *TLR4* and related genes with the incidence of clinical malaria (p = 0.0005). These additional genes include *ABO*, *CAT*, *CD14*, *CD36*, *CR1*, *G6PD*, *GCLM*, *HP*, *IFNG*, *IFNGR1*, *IL13*, *IL1A*, *IL1B*, *IL4R*, *IL4*, *IL6*, *IL13*, *MBL*, *MNSOD*, and *TLR2*. Of specific interest, the previously identified *TLR4* SNP rs4986790 and the novel finding of TRL4 SNP rs5030719 were associated with primary cases of clinical malaria.

**Conclusions:**

These findings highlight a potential central role of TLR4 in clinical malarial pathogenesis. This supports the current literature and suggests that further research into the role of *TLR4*, as well as associated genes, in clinical malaria may provide insight into treatment and drug development.

**Supplementary Information:**

The online version contains supplementary material available at 10.1186/s12936-023-04549-8.

## Background

Malaria is a mosquito-borne communicable disease caused by protists of the *Plasmodium* genus, particularly *Plasmodium falciparum* and *Plasmodium vivax*, which are the two species with a wider health impact. The disease affected approximately 241 million humans worldwide in 2020—a number comparable to that of the COVID-19 global pandemic [1, 2]. Malaria causes severe morbidity and mortality in sub-Saharan Africa, where it is endemic, as well as in South East Asia, Central America, South America, South Asia, and the Eastern Mediterranean Region (the so-called ‘malaria belt’) [1]. It is a leading cause of death in children below 5 years of age in the sub-Saharan region, causing approximately 627,000 deaths in 2020 [1]. Although the parasite has a relatively slow evolutionary rate compared to other infectious agents, mounting anti-parasitic resistance has rendered empirical treatment progressively ineffective, including classical treatments such as quinine or chloroquine [3, 4], prophylactic doxycycline, and the more potent artemisinin [3–7], the bases of most current treatment combinations utilised globally. Indeed, the resistance of *Plasmodium* spp. to artemisinin is an emerging concern for the treatment of malaria particularly in Southeast Asia and China [4–7], with the potential of conferring resistance to parasite populations in India and Africa. Recently, the RTS,S vaccine has been endorsed by the World Health Organization (WHO) as a candidate malaria vaccine, though its efficacy has yet to be established and may be suspect to various genetic and environmental influences [8]. Control of mosquito vectors has provided respite in affected areas, however, the methods of control beyond the use of insecticide-treated bed nets are costly, inefficient, require complex genetic manipulation techniques, and/or utilise chemicals that are harmful to humans, rendering this approach unable to further reduce malaria cases, or contribute towards eradication of disease [9].

The natural host of these parasite species is humans, and because of the selective evolutionary pressures applied by the parasite in its dependency on the human host, many natural genetic resistance mechanisms to malaria have evolved in humans. Traditionally, various well-studied haematologic phenotypes have established mechanisms of defence against *Plasmodium* spp. infection of erythrocytes, namely sickle cell anaemia, β-thalassaemia, glucose-6-phosphate dehydrogenase (G6PD) deficiency, ovalocytosis, and the Duffy antigen negative phenotype [10, 11]. Polymorphisms in other genes from pathways involved in the host immune responses (e.g. cytokine genes) or in parasite pathogenesis (e.g. cytoadhesion to host cells) are likely to impact on the immune response and/or the virulence of the parasite, but evidence of association with malaria has been less robust than that of classical hemoglobinopathies. Genome-wide association studies, notably the MalariaGen Consortium, have attempted to elucidate the associations of genetic variants with malaria, an endeavour which is continuous and periodically produces novel associations [11].

This study utilized a candidate gene approach to identifying genotype-phenotype associations between susceptibility genes and malaria. Of the identified genes, protein-coding genes that played a role in malarial pathogenesis were prioritized for analysis, focusing on those that encoded for cell-surface proteins utilized by *Plasmodium* spp. as adhesion targets [12]. It was hypothesized that some candidate genes are associated with clinical malaria.

Of these, the gene encoding Toll-Like Receptor 4 (TLR4) was found to be of particular interest, and this article focuses on the role TLR plays in the host immune response against *Plasmodium* spp. infection [13–17]. TLR4, also known as CD284, is a transmembrane pattern recognition receptor, whose activation leads to the signalling and production of inflammatory cytokines—a key event in the innate immune response. Regarding malarial infection, this mechanism is predominantly mediated by monocytes, though all myeloid cells have a role to play in the event. The pathogen-associated molecular pattern recognized is *Plasmodium* spp. glycosylphosphatidylinositol (GPI) [13, 18]. The SNP, rs5030719, has previously been associated with diseases such as atherosclerosis [19] and colorectal carcinoma [20], providing context of an immunogenic role; here, its T genotype is reported as a novel risk factor in association with malaria.

It is imperative to understand the aetiology of malaria disease regarding parasite, host, and environment, such that the global burden of malaria can be reduced, or the disease eradicated. To address the hypothesis that candidate genes are associated with clinical malaria, this longitudinal study was conducted to investigate a curated set of human single nucleotide polymorphisms (SNPs) in candidate genes that demonstrated associations between host maternal and children SNPs and early-life malaria susceptibility.

## Methods

### Participants

This study was part of the AgeMal project (ClinicalTrials.gov identifier NCT00231452) described previously [21, 22]. The study was a three-arm randomized, double-blind, placebo-controlled trial carried out in a malaria endemic area (Manhiça district of Southern Mozambique) from August 2005 to July 2009. A total of 349 HIV-negative pregnant women and their new-born babies were recruited and followed up until 2 years after birth, and under continuous morbidity surveillance up to age 4 years. The first exposure to *P. falciparum* was selectively controlled at varying postnatal stages (2 to 5 months, early exposure; 5 to 10 months, late exposure; or none, control) with monthly chemoprophylaxis of sulfadoxine-pyrimethamine and artesunate. However, this strategy did not strongly affect natural acquired immunity and antibody response to *P. falciparum* [21]. Young children were followed up by passive and active malaria case detection for the first 2 years of life to record the incidence of clinical malaria episodes. Passive case detection was done through the morbidity surveillance system at the Manhiça District Hospital, where standardized information on all paediatric outpatient visits and hospital admissions is collected. Active case detection to 10.5 months consisted of weekly home visits by field workers to take axillary temperatures and record any history of fever from the child’s caretaker. From the age of 10.5 to 24 months, these visits were conducted monthly. Anti-malarials were administered if the blood smear reading was positive for asexual *P. falciparum* parasites. As the randomized control trial (RCT) interventions were performed in the child’s first year of life, clinical malaria episodes during the first year were omitted from data analysis. Incidences of primary malarial cases were counted at time-points corresponding to the second, third, and fourth year after a birth; and were defined as having an axillary temperature ≥ 37.5 °C or history of fever within the preceding 24 h plus the presence of *P. falciparum* asexual blood stage parasites of any density when examined under light microscopy. Peripheral blood samples were collected from mothers and infants at delivery, and peripheral blood mononuclear cells (PBMC) were isolated and frozen for future DNA extraction and genotyping [23].

The AgeMal study assessed 349 children, as well as their mothers, over a 2-year period. Subsequent data that were collected yearly until 4 years after birth were included in this study. This number had a steady drop-out of participants until the third yearly data collection, at 285 patients, which then remained approximately the same in the final year. The number of primary malarial cases in each recorded year from the first to fourth respectively totalled 58, 111, 111, and 83 cases; however, the primary clinical malaria data for the first year was omitted as interventions were performed during this time.

Written informed consent was obtained from all mothers. The study was approved by the National Mozambican Ethics Review Committee (Ref: 05/CNBS/05), the Hospital Clínic of Barcelona Ethics Review Committee, and the Princess Margaret Hospital for Children Ethics Committee (1473/EP) in Perth. All experiments were performed in accordance with relevant guidelines and regulations of the Hospital Clínic of Barcelona and the School of Paediatrics and Child Health, University of Western Australia.

### Genotyping

Human genomic DNA was extracted from PBMC using an automated DNA extraction instrument (Autopure LS; Qiagen, Hilden, Germany). Genotyping of the selected functionally important SNPs in immune response and infection resistance related genes was performed by the Australian Genome Research Facility (AGRF) using the iPLEX assay on the MassARRAY system (Sequenom, San Diego, CA) according to the manufacturer’s instructions. All analysed genotypes are listed in Additional file 1: Table S1.

### Data analysis

One hundred and eighty-nine SNPs of interest from the maternal data (95 SNPs) and the children data (94 SNPs) were selected for analysis. Genes previously published from this study in relation with malaria [24, 25] were excluded, leaving a remaining 77 maternal and 74 child SNPs (Additional file 1: Table S1). Candidate SNPs were selected from previously unpublished SNPs in the AgeMal study based on the following rationalia; (1) being associated with malaria or known to be important immune mediators and (2) having a high minor allele frequency in the African population.

The freeware programmes PSPP (https://www.gnu.org/software/pspp/) and R statistics (https://www.r-project.org/), as well as IBM SPSS Statistics 24 (SPSS Inc., Chicago, IL, USA) were used for all statistical analyses.

A Poisson model was used to correlate the number of primary cases (defined as above) with genotypes of SNPs of interest, while using the factors and covariates of: (1) maternal age; (2) parity; (3) child’s sex; (4) use of insecticide-treated nets (ITNs); (5) use of indoor residual spraying (IRS); (6) in utero placental infection (defined by examining histological samples of the placenta); and (7) intervention group. This model treats each genotype category independently (additive model) and was applied to the cumulative data available at the fourth year after birth, which constitutes three time-points corresponding to the children’s ages of 2, 3, and 4 years (the first-year data was omitted due to the influence of maternal immunological protection and administering of interventions during that period). For each SNP, the major allele homozygotes present in this study was defined as the reference genotype. To account for child genotypes confounding effects in maternal genotype results, all significant maternal SNPs were subjected to the Poisson analysis once more, this time including the corresponding child SNP as a co-factor. All results were corrected for multiple testing using a false discovery rate (FDR) adjustment.

All SNPs identified via the Poisson regression as being associated with primary clinical malaria cases were analysed together with other SNPs in the same gene as part of a gene-centric haplotype. Haplotypes most likely associated with malaria were identified using the haplotype trend regression (HTR) model available in R [26], and the haplotype relative frequencies were confirmed using PHASE 2.1.1 [27]. Statistical analyses, including calculation of Hardy–Weinberg statistics were conducted using the Hardy Weinberg package available in R [28].

## Results

Results from the Poisson analysis correlating primary incidence of malaria to SNPs of interest that were found to be statistically significant (p-value after FDR ≤ 0.05) for child and maternal SNPs are listed in Tables 1 and 2, respectively. These SNPs were found in 16 genes in the children (Table 1). The genes, with their respective SNP (given as rs values) and amino acid mutation [if relevant, given in square brackets] are listed: *ABO* (rs8176743 [G235S], rs8176746 [L266M], and rs8176747 [G268V and G268A]), *CD14* (rs5744455), *CD36* (rs1049654 and rs3173798), *CR1* (rs10499885), *GCLM* (rs41303970), *HP* (rs5469), *ICAM1* (rs5490, rs5491 [K56R and K56M], and rs5498 [K469E]), *IFNG* (rs2430561), *IL13* (rs1800925), *IL1A* (rs17561 [A114S]), *IL1B* (rs1143634), *IL4R* (rs1805010 [I75X] and rs1805015 [S503P]), *IL4* (rs4889 [P81L and P81R]), *IL13* (rs1800925), *MBL* (rs11003123 and rs11003124), *TLR2* (rs3804099), and *TLR4* (rs5030719 [Q510H]).

**Table 1:** Single nucleotide polymorphisms (SNPs) in children statistically associated with the incidence of clinical malaria using a Poisson model (q < 0.05)

Gene	rs value	Major	Minor	Minor freq	p-value	q-value	OR
*ABO*	rs8176743	G	A*	0.106061	0.0005	0.003333	0.628 (0.511–0.773)
*ABO*	rs8176746	C	A*	0.10678	0.0005	0.003333	0.618 (0.503–0.758)
*ABO*	rs8176747	G	C*	0.104452	0.0005	0.003333	0.600 (0.488–0.739)
*CD14*	rs5744455	C	T*	0.02632	0.011	0.041739	0.591 (0.394–0.886)
*CD36*	rs1049654	A	C*	0.355932	0.0005	0.003333	0.643 (0.552–0.750)
*CD36*	rs3173798	T*	C	0.312925	0.012	0.041739	1.25 (1.05–1.48)
*CR1*	rs10499885	T*	C	0.355219	0.003	0.017143	1.26 (1.08–1.48)
*GCLM*	rs41303970	C*	T	0.30566	0.0005	0.003333	1.49 (1.25–1.78)
*HP*	rs5469	T	A*	0.425676	0.01	0.041739	0.814 (0.696–0.952)
*ICAM1*	rs5490	A	C*	0.267918	0.0005	0.003333	0.689 (0.579–0.820)
*ICAM1*	rs5491	A	T*	0.319113	0.0005	0.003333	0.687 (0.584–0.807)
*ICAM1*	rs5498	A*	G	0.115658	0.0005	0.003333	1.79 (1.32–2.42)
*IFNG*	rs2430561	T	A*	0.198606	0.011	0.041739	0.803 (0.678–0.951)
*IL13*	rs1800925	C*	T	0.337979	0.005	0.025	1.29 (1.08–1.55)
*IL1A*	rs17561	G*	T	0.146127	0.002	0.012308	1.47 (1.15–1.88)
*IL1B*	rs1143634	C*	T	0.117747	0.005	0.025	1.51 (1.13–2.02)
*IL4R*	rs1805010	G	A*	0.429078	0.0005	0.003333	0.675 (0.574–0.794)
*IL4R*	rs1805015	T*	C	0.395904	0.008	0.035556	1.25 (1.06–1.48)
*IL4*	rs4889	G	del*	0.43696	0.007	0.032941	0.713 (0.558–0.912)
*MBL*	rs11003123	A	G*	0.49481	0.0005	0.003333	0.746 (0.640–0.869)
*MBL*	rs11003124	G	T*	0.491409	0.0005	0.003333	0.753 (0.649–0.875)
*TLR2*	rs3804099	C*	T	0.271277	0.012	0.041739	1.29 (1.06–1.57)
*TLR4*	rs5030719	G	T*	0.024648	0.0005	0.003333	0.465 (0.342–0.633)

In this table, ‘rs values’ are as defined in the NCBI dbSNP repository, ‘minor frequency’ denotes the minor allele frequencies of each SNP from the genotypes analysed in this study, p-values are the output of Poisson analysis, and q-values are the p-values adjusted for multiple testing using a false detection rate (FDR) model. OR denotes the odds ratio of clinical malaria for the minor allele compared to the major allele; the subsequent columns of lower CI and upper_CI, respectively denote the lower and upper boundaries of the OR 95% confidence interval. In the columns defining the reference and alternate alleles, the genotype conferring risk to malarial incidence is marked with an asterisk (*) as inferred from the OR and its confidence interval

**Table 2:** Single nucleotide polymorphisms (SNPs) in mothers statistically associated with the incidence of clinical malaria using a Poisson model (q < 0.05)

Gene	rs_value	Reference	Alternate	Minor frequency (mother)	Minor frequency (child)	p-value	Adjusted p-value	q-value	OR
*CAT*	rs769214	A*	G	0.479094	0.45993	0.0005	0.0005	0.005	1.39 (1.20–1.61)
*G6PD*	rs1050829	A*	G	0.311224	0.282759	0.004	0.0005	0.029091	1.30 (1.09–1.56)
*HP*	rs5470	C*	G	0.096939	0.101375	0.0005	0.003	0.005	1.82 (1.39–2.39)
*ICAM1*	rs5498	A	G*	0.123746	0.115658	0.003	0.0005	0.024	0.705 (0.561–0.884)
*IFNGR1*	rs2234711	T	C*	0.456376	0.453767	0.0005	0.0005	0.005	0.664 (0.573–0.769)
*IFNGR1*	rs7749390	A	G*	0.45	0.452862	0.0005	0.0005	0.005	0.661 (0.571–0.765)
*IFNG*	rs2430561	T	A*	0.201754	0.198606	0.002	0.004	0.017778	0.734 (0.612–0.893)
*IL6*	rs2069830	C	T*	0.13255	0.142612	0.0005	0.0005	0.005	0.635 (0.531–0.760)
*MNSOD*	rs4880	T	C*	0.434564	0.423469	0.0005	0.0005	0.005	0.717 (0.619–0.830)
*TLR2*	rs7656411	G*	T	0.333904	0.333333	0.0005	0.0005	0.005	1.51 (1.29–1.78)
*TLR4*	rs5030719	G	T*	0.02489	0.024648	0.0005	0.0005	0.005	0.421 (0.301–0.588)

In this table, ‘rs values’ are as defined in the NCBI dbSNP repository, ‘minor frequency’ columns denote the minor allele frequencies of each SNP from the genotypes analysed in this study respectively for mother and child, p-values are the output of Poisson analysis, adjusted p-values are the corresponding p-values re-calculated to consider the children’s SNP as a co-factor, and q-values are the p-values adjusted for multiple testing using a false detection rate (FDR) model. OR denotes the odds ratio of clinical malaria for the minor allele compared to the major allele; the subsequent columns of lower_CI and upper_CI, respectively denote the lower and upper boundaries of the OR 95% confidence interval. In the columns defining the reference and alternate alleles, the genotype conferring risk to malarial incidence is marked with an asterisk (*) as inferred from the OR and its confidence interval

Similarly, maternally associated SNPs were found in 10 genes (Table 2). The genes, with their respective SNP (given as rs values) and amino acid mutation [if relevant, given in square brackets] are listed: *CAT* (rs769214), *G6PD* (rs1050829 [N156Y and N156D]), *HP* (rs5470), *ICAM1* (rs5498 [K469E]), *IFNGR1* (rs2234711 and rs7749390), *IFNG* (rs2430561), *IL6* (rs2069830 [P32S]), *MNSOD* (rs4880 [V16A]), *TLR2* (rs7656411), and *TLR4* (rs5030719 [Q510H]), all of which were still statistically significant after including in the analysis the respective children’s alleles as co-factors. An overlapping set of genes, *HP*, *ICAM1*, *IFNG*, *TLR2*, and *TLR4* were found to contain SNPs statistically significantly associated with malaria in both maternal and child analyses. Additionally, the SNPs rs5498 in *ICAM1*, rs2430561 in *IFNG*, and rs5030719 in *TLR4* were found in both maternal and child analyses, whereas the other SNPs in *HP*, and *TLR2* differed between mother and child. In both cases of *IFNG* rs2430561 and *TLR4* rs5030719, the risk genotypes in maternal and child analyses were found to be the same. The T variant of SNP rs5030719 as a malarial risk factor is a novel finding previously unreported in the literature. As a measure of quality control, the allelic distribution of all genes was assessed in relation to Hardy–Weinberg equilibrium (Additional file 2: Table S2 and Additional file 3: Table S3). Of the SNPs identified, rs1050828 and rs1050829 in child *G6PD*, and rs4696483 in maternal *TLR2* did not adhere to the Hardy–Weinberg equilibrium expectation, and so were omitted from further analysis.

Haplotype trend regression of the SNPs identified in Tables 1 and 2 identified five child haplotypes in three genes as being associated with primary cases of clinical malaria: *ABO* (CAA and GCG), *IL13* (GCACT and GTGCT), and *TLR4* (AT); as well as four haplotypes in three maternal genes, *IFNGR1* (AT(TT) and GC(TT)), *IL6* (GCT), and *TLR4* (GT). The SNP rs values and further information on the haplotypes are provided in Table 3. *TLR4* was the only gene identified to be statistically associated with malaria in both child and maternal analysis as well as producing haplotypes associated with the disease in the HTR analysis.

**Table 3:** Genes and their haplotypes that are statistically associated with the incidence of clinical malaria via haplotype trend regression (HTR) analysis

Gene	Maternal/child	Haplotype	f-statistic	p-value	rs_value	Chromosome	Position (hg38)
*ABO*	Child	CAA	4.984505	0.028	rs8176747; rs8176746; rs8176743	9	133255928; 133255935; 133256028
*ABO*	Child	GCG	4.984505	0.028	rs8176747; rs8176746; rs8176743	9	133255928; 133255935; 133256028
*IFNGR1*	Maternal	AT(TT)	2.653187	0.073	rs7749390; rs2234711; rs41401746	6	137219233; 137219383; 137219797–137219798
*IFNGR1*	Maternal	GC(TT)	2.653187	0.073	rs7749390; rs2234711; rs41401746	6	137219233; 137219383; 137219797–137219798
*IL13*	Child	GCACT	1.090957	0.373	rs2069739; rs1800925; rs2069743; rs2066960; rs20541	5	132656916; 132657117; 132657583; 132658743; 132660272
*IL13*	Child	GTGCT	1.090957	0.373	rs2069739; rs1800925; rs2069743; rs2066960; rs20541	5	132656916; 132657117; 132657583; 132658743; 132660272
*IL6*	Maternal	GCT	4.347798	0.018	rs1800796; rs1800795; rs2069830	7	22726627; 22727026; 22727518
*TLR4*	Maternal	GT	3.42998	0.027	rs4986790; rs5030719	9	117713024; 117713658
*TLR4*	Child	AT	3.734751	0.021	rs4986790; rs5030719	9	117713024; 117713658

The ‘maternal/child’ column defines if the gene and haplotype was identified in the maternal or children genomes, the ‘haplotype’ column lists the alleles found in the haplotype in sequence of increasing genomic position, further defined in the columns ‘rs_value’ (as defined in the NCBI dbSNP repository), ‘chromosome’, and ‘position’ (as annotated in human whole genome build hg38). The ‘f-statistic’ and ‘p-value’ columns respectively detail the Fisher-statistic for goodness-of-fit of the haplotype to the tested model and haplotype trend regression (HTR) p-value for association of the haplotype with primary cases of clinical malaria. The two haplotypes for *IFNGR1* involve rs41401746, which represents a dinucleotide polymorphism, and is represented here in brackets (TT). Further description is given in Additional file 1: Table S1

## Discussion

Primary analysis of these targeted genetic polymorphisms highlights a shortlist of genes that are potentially associated with malaria and have biological plausibility in pathology of the disease. Of these, the only gene identified in both maternal and child analyses was *TLR4*. Further HTR analysis implicates the additional genes *ABO, IFNGR1, IL13*, and *IL6.*

The identification of various interleukin (*IL*) and toll-like receptor (*TLR*) genes is expected, as in the literature it has been demonstrated that these immunologically related molecules are associated with clinical malaria, ranging across the spectrum of impact from cellular biology to clinical drug and vaccine development [18]. Befitting any general immune response mechanism, various cytokines may potentially modulate *ICAM1* expression, of which we have found evidence for statistical association of *IL1*, *IL4* (and its receptor, *IL4R*), *IL6*, *IL13, TLR2, TLR4*, and *IFNG* (and its receptor, *IFNGR1*) polymorphisms with malaria. In this context, it can be argued that these immunogenically related genes, along with the identified gene *HP*, play a supporting role in inducing *ICAM1*. Circulating IL6 and IFN-γ levels are associated with clearance of the parasite, though in high concentrations, may manifest as extreme symptoms of malaria [29].

Noteworthy amongst these genes is *TLR4*, which have been identified in all analyses described above (Tables 1 and 2, and 3). Supporting literature has also identified this gene as integral in the pathological response to malaria [13, 15], and is demonstrable as an anti-malarial vaccine target [14]. TLRs, including TLR4 identified herein, are part of the innate immune system, being expressed primarily by monocytes and playing a role in recognition of malarial molecular markers, in particular GPI, ostensibly implicating variations in TLR4 with the elicited immune response [18]. Polymorphisms in the SNP rs4986790 (Asp299Gly or 896 A/G), which was detected in our HTR analysis is also extensively studied and associated with malaria [16, 17], although our findings did not discern a difference between either A or G variants (Table 3). The G allele of rs5030719 is identified as a novel risk factor for clinical malaria, identified in children, as well as in mothers after adjusting for child genotype influence.

Intermediary immunomodulators, such as TLRs, including TLR4, bridge signalling between Pathogen-Associated Molecular Patterns (PAMPs) and intercellular adaptors such as MyD88 and TIR domain-containing adapter-inducing IFN-β (TRIF), which then have a plethora of intercellular effects, including cytokine signalling [30]. An agnostic hypothesis of observing involvement of TLR4 in the malarial defence signalling pathway would be the expectation of numerous cytokines, as observed in this study, however this cannot be statistically correlated with the limited data compared to the prodigious number of potential candidate genes. The observed results identifying interleukins (*IL4*, *IL4R*, *IL6*, and *IL13*), interferons (*IFNG* and *IFNGR1*), and downstream immunomodulatory proteins (*CD14* and *CD36*) fit the biological narrative of this hypothesis, warranting future interrogation into the role and potential use of these cytokines in treatment of malaria.

ICAM-1 is a cell-surface receptor utilized by *Plasmodium* spp. mature trophozoites and schizonts in cytoadhesion between parasitized red blood cells and endothelial cells lining the local microvasculature, leading to parasite sequestration. This interaction is mediated by the parasitic ligand, *P. falciparum* erythrocyte membrane protein 1 (PfEMP-1) and possibly other parasite-related proteins [31]. Although the details of this process are obscure, there is a demonstrable reduction in clinical symptoms when host ICAM-1 expression is downregulated, suggesting a key role of ICAM-1 in malarial pathogenesis [32–35]. There is strong literature evidence for *ICAM1* [34, 36–40], as well as another gene identified herein, *GCLM*, and its associated gene, *GCLC* [24] being potential molecular mediators for the pathology of malaria. There are, therefore, potential targets for malaria prevention and treatment, however, the mechanisms of involvement of these genes in malarial pathogenesis is yet unclear. Expression levels of *ICAM1* are low in peripheral blood, but they are expressed at higher levels in the liver [41], suggesting that the expression of alleles of these genes may mediate an effect on the maturation of the malarial schizont stage, which takes place in the liver. To expand on the role of *GCLC* and *GCLM* in malaria pathology, the glutathione synthesis pathway has a known role in combating clinical malaria (and infections in general), playing a key role in the scavenging of free radicals and alleviating oxidative stress injury. This in turn allows for better maintenance of cellular homeostasis during infection periods, reducing the incidence of cellular damage and consequently, apoptosis [42]. Much of the biochemical knowledge of the glutathione pathway, however, is limited to generalized cellular damage, and the specific role it plays in malarial infections remains to be uncovered.

Interestingly, the *ICAM1* SNPs, rs5490, rs5491, and rs5498, have been published as being associated with malaria [33, 34, 36–40, 43], a finding which was later scrutinized in a larger meta-analysis, which could not corroborate the earlier findings [44]. Further examination of the risk alleles of the *ICAM1* SNPs in association with clinical malaria phenotypes (Additional file 4: Table S4), suggests that maternal mutations in rs5490 (A>C) and rs5491 (A>T) increased the risks of malarial anaemia odds ratios of 3.027 and 2.043, respectively). The *ICAM1* SNP rs5698 polymorphism produced conflicting results, where the minor polymorphism (A) in children was observed to confer risk (OR = 1.78783, 95% CI [1. 31,785, 2.42298]), whereas the major polymorphism (G) in mothers was observed to confer risk (OR = 0.704688, 95% CI [0.561019, 0.884264]). The relationship between genotype and phenotype here may be spurious, or potentially complex, requiring more specialized further studies. The literature supporting this association is mixed, however, more recent publications suggest there is no association between malaria and genotypes of rs5498 [44, 45].

In these analyses, each polymorphism was analyzed using an additive model; a co-dominant model was also generated, but data not reported here. It is biologically plausible that, like many protective polymorphisms, the heterozygous genotypes have attained a skewed Hardy–Weinberg equilibrium in the population, and confer a phenotypic advantage against infection, which amounts to a small effect size [46]. Existing molecular evidence on the pathology and binding capacity of *Plasmodium* spp. parasites to variant *ICAM1* molecules (*ICAM1*^Kilifi^) further supports this observation [47], however, it is worth noting that the constant co-evolution of host and parasite may marginalize the observation of such pronounced evidence in in vitro settings. Although cytokine modulation of ICAM-1 expression is documented in the literature in specific or niche contexts, none have suggested an association pertaining to clinical malaria [43].

Additionally, it is worth mentioning is that examination of the *ABO* gene provides evidence of risk factors predisposing to malarial infection in rs8176743 and rs8176746. The literature suggests that O blood phenotype is protective against malaria, though we could not correlate our findings with definitive blood typing. A possible mechanism of action is that infected O erythrocytes are more easily cleared than A or B, having reduced cytoadhesion compared to the A phenotype [48]. This is further reinforced by the observation that the O phenotype is more prevalent in areas in which malaria is endemic. To further expound the reduced cytoadhesion mechanism in preventing malaria, other genes that produce or enhance this phenotype are also found to confer protection against malaria, namely *ICAM1* and *CD36*, both of which are identified herein.

Taken in context, these findings complement the body of knowledge regarding malarial infection and host defence, while providing further information on candidate genes for future research. The list of genes analysed in this study (and previously published, related studies), is not fully comprehensive, as it includes genes involved in the innate immune system and does not include niche genes such as those coding for sporozoite adhesion proteins, specific natural killer cell receptors, or expression of variable components of the adaptive immunity. However, a global interpretation of host risk genes here portrays a succinct and plausible narrative in that variations in major defence and signalling pathways involved in malarial infection are statistically associated as risk factors for clinical malaria outcomes and can, therefore, be targeted in future research for developing treatments and vaccines against malaria.

## Conclusion

In conclusion, this study has utilized a comprehensive randomized control clinical trial to create a longitudinal dataset following maternal and children development to ascertain potential relationships between incidence of primary malaria cases with select candidate genes known to play a role in malarial pathogenesis. The results herein provide evidence for the association of *TLR4* and related genes, *ICAM1*, *IFNGR1, IL13*, and *IL6*, as well as *ABO*, with the incidence of clinical malaria. In particular, the variations of T in the SNP rs5030719 of *TLR4* conferred risk of clinical malaria compared to the G allele. This supports the current literature pertaining to the disease, as well as suggests that further research into the role of *TLR4*, as well as other related genes, in pathogenesis of clinical malaria may provide further insight into drug development and treatment of the disease.

## Supplementary Information

**Additional file 1: Table S1.** Table of genes and single nucleotide polymorphismsanalysed in this study.

**Additional file 2: Table S2.** Hardy–Weinberg equilibrium statistical values for child single nucleotide polymorphismsstudied herein. Only SNPs with a minor allele count of more than 5 were included in this analysis. The column for ‘chi-square’ values is calculated with 1 degree of freedom. The column for ‘p-value’ includes a continuity correction. Column ‘D’ denotes the disequilibrium coefficient for each SNP.

**Additional file 3: Table S3.** Hardy–Weinberg equilibrium statistical values for maternal single nucleotide polymorphismsstudied herein. Only SNPs with a minor allele count of more than 5 were included in this analysis. The column for ‘chi-square’ values is calculated with 1 degree of freedom. The column for ‘p-value’ includes a continuity correction. Column ‘D’ denotes the disequilibrium coefficient for each SNP.

**Additional file 4: Table S4.** Table of statistically significant single nucleotide polymorphismsand insertion/deletion eventscorrelated with episodes of anaemia or *Plasmodium* sp. Parasitaemia in child and/or mother. The column ‘rs_value’ lists the rs number associated with the SNP as defined in the NCBI dbSNP repository, ‘maternal/child’ lists the genome in which the SNP was identified, and ‘association’ lists the phenotype statistically associated with the SNP. Statistical columns respectively list the beta value for the association, standard error, Wald-value for hypothesis-testing, the p-value for the test, exponent of the beta value’), as well as the upper and lower values for the 95% confidence interval limit. The final columns, ‘ref’ and ‘alt’ denote the reference and alternate genotypes for SNPs and indels.

## Data Availability

The datasets generated and analysed during the current study are not publicly available due to patient confidentiality and privacy but are available from the corresponding author on reasonable request. The subset of genetic and statistical data relevant to the analysis herein are provided in Additional tables.

## Abbreviations

AECID: Spanish Agency for International Cooperation and Development
AGRF: Australian Genome Research Facility
FDR: False detection rate
G6PD: Glucose-6-phosphate dehydrogenase
GPI: Glycosylphosphatidylinositol
IFN-β: Interferon β
IL: Interleukin
ITN: Insecticide-treated net
PAMP: Pathogen-associated molecular pattern
PBMC: Peripheral blood mononuclear cells
PfEMP-1: *P. falciparum* erythrocyte membrane protein 1
RCT: Randomized Controlled Trial
SNP: Single nucleotide polymorphism
TLR: Toll-like receptor
TRIF: TIR domain-containing adapter-inducing IFN-β
